# Bidirectional Effects of Cannabidiol on Contextual Fear Memory Extinction

**DOI:** 10.3389/fphar.2016.00493

**Published:** 2016-12-16

**Authors:** Chenchen Song, Carl W. Stevenson, Francisco S. Guimaraes, Jonathan L. C. Lee

**Affiliations:** ^1^School of Psychology, University of BirminghamBirmingham, UK; ^2^School of Biosciences, University of Nottingham, Sutton Bonington CampusLoughborough, UK; ^3^Department of Pharmacology, University of São PauloSão Paulo, Brazil

**Keywords:** memory, extinction, fear, contextual, cannabinoid

## Abstract

Cannabidiol (CBD) has been established to have both acute and long-lasting effects to reduce fear memory expression. The long-lasting impact might be mediated by an enhancement of memory extinction or an impairment of memory reconsolidation. Here, we directly compared the effects of i.p. injections of cannabidiol (10 mg/kg) with those of the NMDA receptor antagonist MK-801 (0.1 mg/kg) and partial agonist D-cycloserine (DCS; 15 mg/kg) in order to determine the mnemonic basis of long-term fear reduction. We showed that under conditions of strong fear conditioning, CBD reduced contextual fear memory expression both acutely during the extinction session as well as later at a fear retention test. The latter test reduction was replicated by DCS, but MK-801 instead elevated test freezing. In contrast, when initial conditioning was weaker, CBD and MK-801 had similar effects to increase freezing at the fear retention test relative to vehicle controls, whereas DCS had no observable impact. This pattern of results is consistent with CBD enhancing contextual fear memory extinction when the initial conditioning is strong, but impairing extinction when conditioning is weak. This bidirectional effect of CBD may be related to stress levels induced by conditioning and evoked at retrieval during extinction, rather than the strength of the memory *per se*.

## Introduction

Cannabidiol (CBD) is the major non-psychotropic constituent of the *Cannabis* plant and has anxiolytic therapeutic potential. Acute administration of CBD decreases the expression of contextual conditioned fear memories in rats (Resstel et al., 2006; Lemos et al., 2010; but see Marinho et al., 2015) as well as reducing physiological fear-related responses in humans (Fusar-Poli et al., 2009). These acute effects may be mediated by a variety of central loci, including the amygdala (Fusar-Poli et al., 2009), prelimbic cortex (Lemos et al., 2010), and bed nucleus of the stria terminalis (Gomes et al., 2012). Moreover, the fear-reducing impact of CBD is likely to be via its action as a 5-HT1A partial agonist (Gomes et al., 2012; Fogaça et al., 2014).

CBD can also result in long-lasting fear reduction, when combined with either extinction training or brief memory reactivation/destabilization to impair reconsolidation (Bitencourt et al., 2008; Stern et al., 2012; Das et al., 2013; Do Monte et al., 2013; Gazarini et al., 2015). Infusion of CBD intra-cerebroventricularly (Bitencourt et al., 2008) or into the infralimbic cortex (Do Monte et al., 2013) prior to extinction training in rats enhanced the subsequent reduction in contextual fear expression indirectly via CB1 receptor activation. Moreover, CBD also enhanced the beneficial impact of extinction training in a human fear conditioning setting (Das et al., 2013). Similarly, CBD might diminish fear expression when combined with brief memory reactivation and the engagement of destabilization/reconsolidation processes. Systemic injection of CBD immediately after brief memory retrieval resulted in a long-lasting subsequent impairment in contextual freezing (Stern et al., 2012). This effect was dependent upon CB1R activity and was also observed in a model of post-traumatic stress disorder, albeit necessitating pharmacological enhancement of memory destabilization (Gazarini et al., 2015).

The apparent effect of CBD both to enhance extinction and impair reconsolidation is of particular note, given that other treatments have a common impact on both processes, thereby leading to bidirectional effects on fear expression (Pedreira and Maldonado, 2003; Lee et al., 2006; Tronson et al., 2006; Schramm et al., 2016), or are selective for only one of the processes (Suzuki et al., 2004; de la Fuente et al., 2011; Tronson et al., 2012). However, reconsolidation impairments are not easily distinguished from potentiation of extinction (e.g., Trent et al., 2015). Therefore, it remains unclear whether the aforementioned studies do indeed reflect distinct mechanisms of action to produce similar reductions in fear, especially given the common dependence upon CB1Rs. While hippocampal CB1R antagonism by AM251 appears to produce the opposite effects to CBD (de Oliveira Alvares et al., 2008), intra-amygdala infusion of AM251 impairs, rather than enhances, fear memory reconsolidation (Ratano et al., 2014). However, hippocampal CB1/2R agonism does impair contextual fear memory reconsolidation (Santana et al., 2016). With systemic administration, CB1R antagonism has been shown previously to impair contextual fear memory extinction (Suzuki et al., 2004) and memory destabilization (Suzuki et al., 2008), whereas CB1R agonists enhance destabilization without reducing contextual freezing alone (Lee and Flavell, 2014). Therefore, the somewhat mixed picture concerning CB1R involvement in reconsolidation and extinction has implications for the interpretation of CB1R-dependent CBD effects on contextual fear.

A further complication is the lack of no-extinction control groups in the CBD extinction studies (Bitencourt et al., 2008; Das et al., 2013; Do Monte et al., 2013). While not as routinely implemented as in reconsolidation studies, no-extinction controls are important to rule out potential direct long-lasting effects of CBD upon the subsequent test. This is especially pertinent as CBD has been shown to reduce cue-induced heroin seeking when given 24 h, but not 30 min, prior to the test (Ren et al., 2009), and has long-lasting anti-depressant effects up to 14 d post-injection (Linge et al., 2016). Therefore, here we revisited the putative effect of CBD to enhance contextual fear memory extinction, with the addition of no-extinction controls. Moreover, we compared the effect of CBD against the impact of systemic administration of MK-801 and D-cycloserine (DCS), given that NMDA receptor antagonism and partial agonism have been established to impair (Suzuki et al., 2004) and enhance (Yamada et al., 2009, 2011) context fear memory extinction, respectively.

## Materials and methods

### Subjects

100 experimentally-naïve male Lister Hooded Rats (Charles River, UK; aged 7–8 weeks at the start of experimental procedures) were housed in quads under a 12 h light cycle (lights on at 0700) in a specialist animal facility. Individually-ventilated cages contained aspen chip bedding and a plexiglass tunnel for environmental enrichment. Rats had free access to food and water other than during behavioral sessions. Experiments took place between 0900 and 1200 in a behavioral laboratory. At the end of the experiment, animals were humanely killed using a rising concentration of CO2. All procedures were approved by the local animal welfare and ethical review board and carried out in accordance with the United Kingdom 1986 Animals (Scientific Procedures) Act, Amendment Regulations 2012 (PPL 70/7662).

### Drugs

Drugs were administered intraperitoneally (1 ml/kg) 30 min prior to extinction sessions. CBD (THC pharm, Germany; 10 mg/kg) was dissolved in DMSO and diluted to a final vehicle of 20% DMSO in Saline with 0.1% Tween 80 immediately prior to use. MK-801 (Sigma, UK; 0.1 mg/kg) and D-cycloserine (DCS; Sigma, UK; 15 mg/kg) were dissolved in saline vehicle and stored at −10°C until use. Doses of drugs were taken from published literature showing modulatory effects on fear memory (Lee et al., 2006; Stern et al., 2012).

### Behavioural procedures

All behavioral procedures were carried out in conditioning chambers (MedAssociates, VT) as previously described (Lee and Hynds 2013), with freezing behavior automatically recorded by Videotracking software (Viewpoint Life Sciences, France). All rats received the same behavioral training, apart from a single difference in the conditioning session.

One day prior to conditioning, rats were exposed twice to the conditioning chamber for 10 min on each occasion, with a 60-min ITI. Conditioning consisted of either a 7-min or a 10-min session for weak and strong conditioning, respectively. For both conditions, there was a 5-min pre-shock period, followed by exposure to a sequence of 0.5-mA, 1-s footshocks at 300 s and 341 s (as well as 402, 423, 484, and 525 s for the strong conditioning). On the next day, rats were given i.p. injections 30 min prior to a 20-min re-exposure to the context (extinction session). Finally, the day after extinction, rats were tested in a 5-min context re-exposure.

### Statistical analyses

Data are presented as mean + SEM. Contextual freezing at test was analyzed in JASP (JASP Team, 2016) using 2-way ANOVA for a direct comparison of extinction and no-extinction conditions, followed by planned one-way ANOVA comparisons of drug effects within each condition. The extinction sessions (sub-divided into four 5-min bins) were analyzed by mixed 2-way ANOVA. Mauchly's W was used as a test of sphericity, and the Greenhouse-Geisser correction employed when the assumption of sphericity was violated. $\eta_{p}^{2}$ was used as an estimate of effect size and BF_10_ is also reported as the outcome of Bayesian analyses for the estimation of posterior probability (Jarosz and Wiley, 2014).

## Results

**Figure 1:**
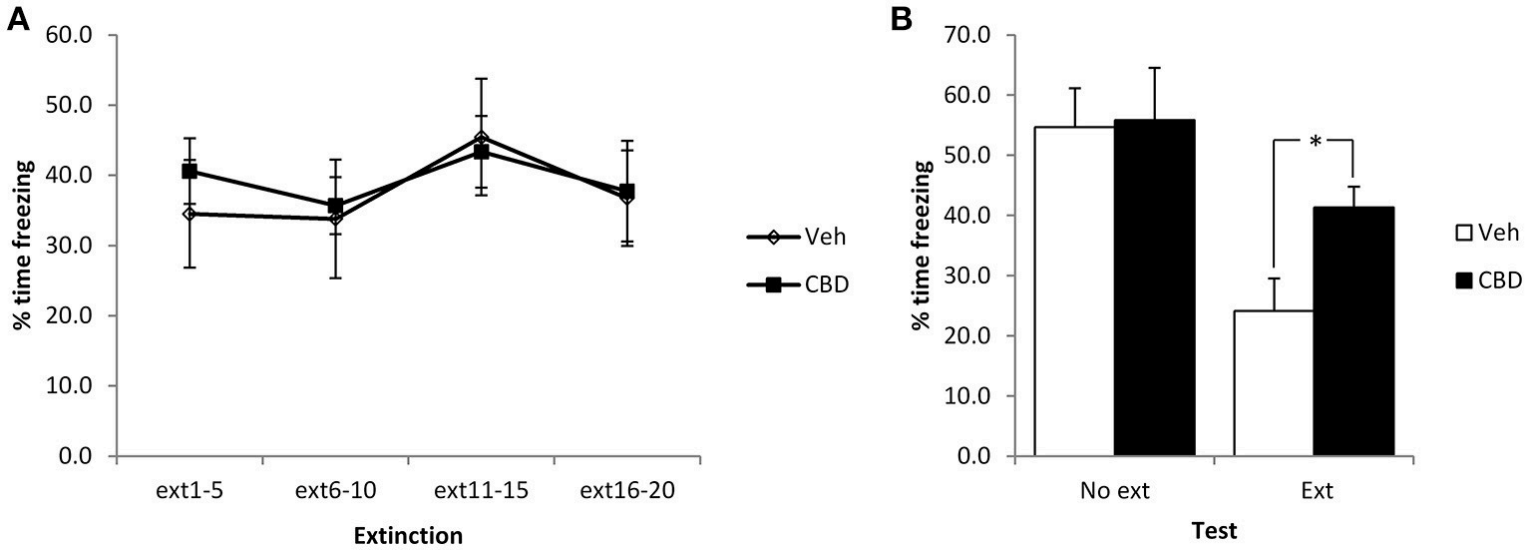
**Pre-extinction CBD attenuates the subsequent decline in contextual freezing following weak conditioning**. CBD was injected 30 min prior to the extinction session, or in the absence of an extinction session. There were no acute effects of CBD during the extinction session itself **(A)**, but there was an extinction-dependent effect at the subsequent test **(B)**. Vehicle-injected rats froze at lower levels than CBD-treated rats. *N* = 6–8 per group. ^*^p < 0.05.

We initially used a mild conditioning procedure that was similar in intensity to our previous work. With two mild footshocks, a moderate level of contextual freezing (35–40%) was observed at the start of the extinction session. In comparing the extinction and no-extinction conditions (Figure 1), while there was a significant effect of the extinction session [*F*_(1, 23)_ = 14.1, *p* = 0.001, $\eta_{p}^{2}$ = 0.38, BF_10_ = 37.7], there was no effect of CBD [*F*_(1, 23)_ = 2.4, *p* = 0.14, η^2^_*p*_ = 0.09, BF_10_ = 0.91] or CBD x extinction interaction [*F*_(1, 23)_ = 1.8, *p* = 0.19, η^2^_*p*_ = 0.07, BF_10_ = 0.84].

Planned comparison of the extinction condition revealed a significant effect of CBD to elevate contextual freezing at test [*F*_(1, 12)_ = 7.1, *p* = 0.02, η^2^_*p*_ = 0.37, BF_10_ = 3.2], but no observable acute effect on contextual freezing during the extinction session [CBD: *F*_(1, 12)_ = 0.11, *p* = 0.74, η^2^_*p*_ = 0.009, BF_10_ = 0.43; CBD x bin: *F*_(3, 36)_ = 0.28, *p* = 0.84, η^2^_*p*_ = 0.02, BF_10_ = 0.23]. Surprisingly, there was no evidence for within-session extinction [*F*_(3, 36)_ = 1.7, *p* = 0.19, η^2^_*p*_ = 0.12, BF_10_ = 0.69]. In the no-extinction control condition, planned comparisons revealed no effect of CBD at test [*F*_(1, 11)_ = 0.013, *p* = 0.91, η^2^_*p*_ = 0.001, BF_10_ = 0.46]. Therefore, CBD appears to impair the normal reduction in contextual freezing observed following extinction training in an effect that is not attributable to direct effects of CBD injection upon test performance on the next day.

**Figure 2:**
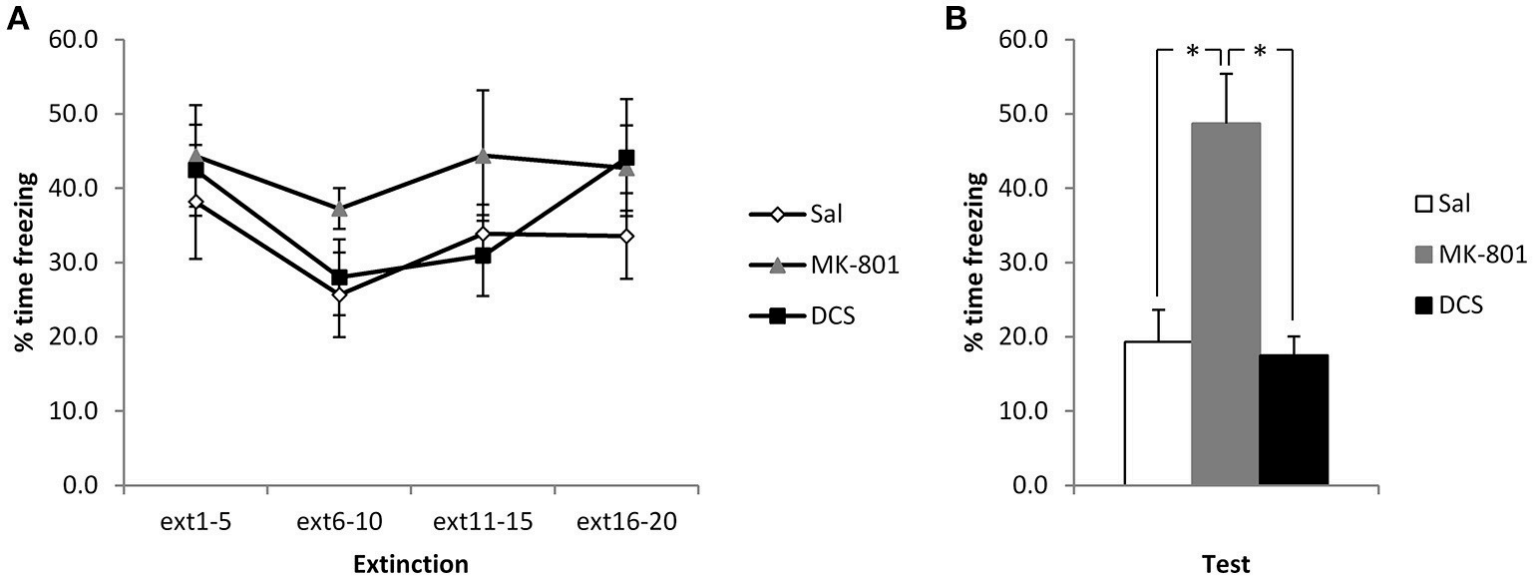
**Pre-extinction MK-801 attenuates the subsequent decline in contextual freezing following weak conditioning**. MK-801 and DCS were injected 30 min prior to the extinction session. There were no acute effects of either drug during the extinction session itself **(A)**. MK-801, but not DCS, increased contextual freezing at the subsequent test **(B)**. *N* = 6–7 per group. ^*^p < 0.05.

In order to disambiguate whether the CBD-induced elevation of freezing reflects an impairment of extinction or perhaps an enhancement of reconsolidation, we administered MK-801 and DCS under the same experimental conditions. Injection of MK-801 appeared to impair the extinction of contextual freezing, whereas DCS had no observable effect (Figure 2). ANOVA revealed a significant difference between the groups [*F*_(2, 17)_ = 14.6, *p* < 0.001, η^2^_*p*_ = 0.63, BF_10_ = 125], with *post-hoc* comparisons (*p* < 0.05) showing that the MK-801 group displayed higher levels of contextual freezing than both the Saline control group and the DCS-injected rats (the latter two groups not differing from each other). This difference at test was not attributable to pre-existing differences at the extinction session, with no effect of group [*F*_(2, 17)_ = 1.30, *p* = 0.30, η^2^_*p*_ = 0.13, BF_10_ = 0.57] or group x bin [*F*_(3.6, 30.4)_ = 0.67, *p* = 0.60, η^2^_*p*_ = 0.07, BF_10_ = 0.16] interaction. While there was a significant effect of bin [*F*_(1.8, 30.4)_ = 3.88, *p* = 0.036, η^2^_*p*_ = 0.19, BF_10_ = 3.90], this was not driven by a decline in freezing over the course of the session, and so there was again little evidence for within-session extinction.

**Figure 3:**
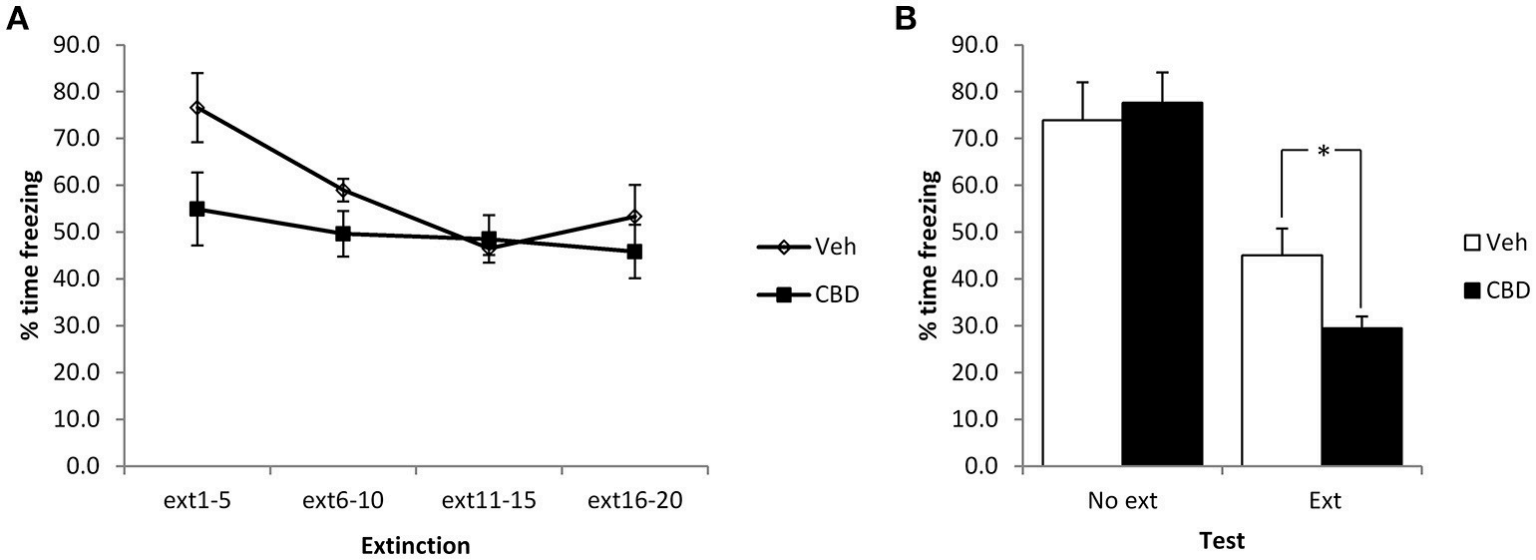
**Pre-extinction CBD potentiates the subsequent decline in contextual freezing following strong conditioning**. CBD was injected 30 min prior to the extinction session, or in the absence of an extinction session. CBD acutely reduced contextual freezing during the extinction session **(A)**, as well as enhancing the extinction-dependent reduction of freezing at the subsequent test **(B)**. *N* = 7 per group. ^*^p < 0.05.

Given the unexpected effects of CBD observed above, in terms of our failure to replicate the reduction in contextual freezing observed previously, the lack of acute effect of CBD at the extinction session and the lack of within-session extinction, we repeated the experiment using stronger conditioning parameters involving 6 mild footshocks. In comparing the extinction and no-extinction conditions (Figure 3), while there was a significant effect of the extinction session [*F*_(1, 24)_ = 47.4, *p* < 0.001, η^2^_*p*_ = 0.66, BF_10_ = 27.5 × 10^3^], there was no effect of CBD [*F*_(1, 24)_ = 1.1, *p* = 0.30, η^2^_*p*_ = 0.05, BF_10_ = 0.52] or CBD x extinction interaction [*F*_(1, 24)_ = 3.0, *p* = 0.10, η^2^_*p*_ = 0.11, BF_10_ = 1.2]. Planned comparison of the extinction condition revealed a significant effect of CBD to reduce contextual freezing at test [*F*_(1, 12)_ = 7.3, *p* = 0.02, η^2^_*p*_ = 0.38, BF_10_ = 3.3], as well as a moderate acute impairment of contextual freezing during the extinction session [CBD: *F*_(1, 12)_ = 5.4, *p* = 0.04, η^2^_*p*_ = 0.31, BF_10_ = 1.1; CBD x bin: *F*_(3, 36)_ = 1.9, *p* = 0.15, η^2^_*p*_ = 0.16, BF_10_ = 0.90]. Under these conditions there was a significant reduction in contextual freezing during the course of the extinction session [*F*_(3, 36)_ = 5.2, *p* = 0.004, η^2^_*p*_ = 0.30, BF_10_ = 18.3]. In the no-extinction control condition, planned comparisons revealed no effect of CBD at test [*F*_(1, 12)_ = 0.15, *p* = 0.70, η^2^_*p*_ = 0.012, BF_10_ = 0.47]. Therefore, with stronger conditioning CBD appears to enhance the normal reduction in contextual freezing observed following extinction training in an effect that is not attributable to direct effects of CBD injection upon test performance on the next day.

**Figure 4:**
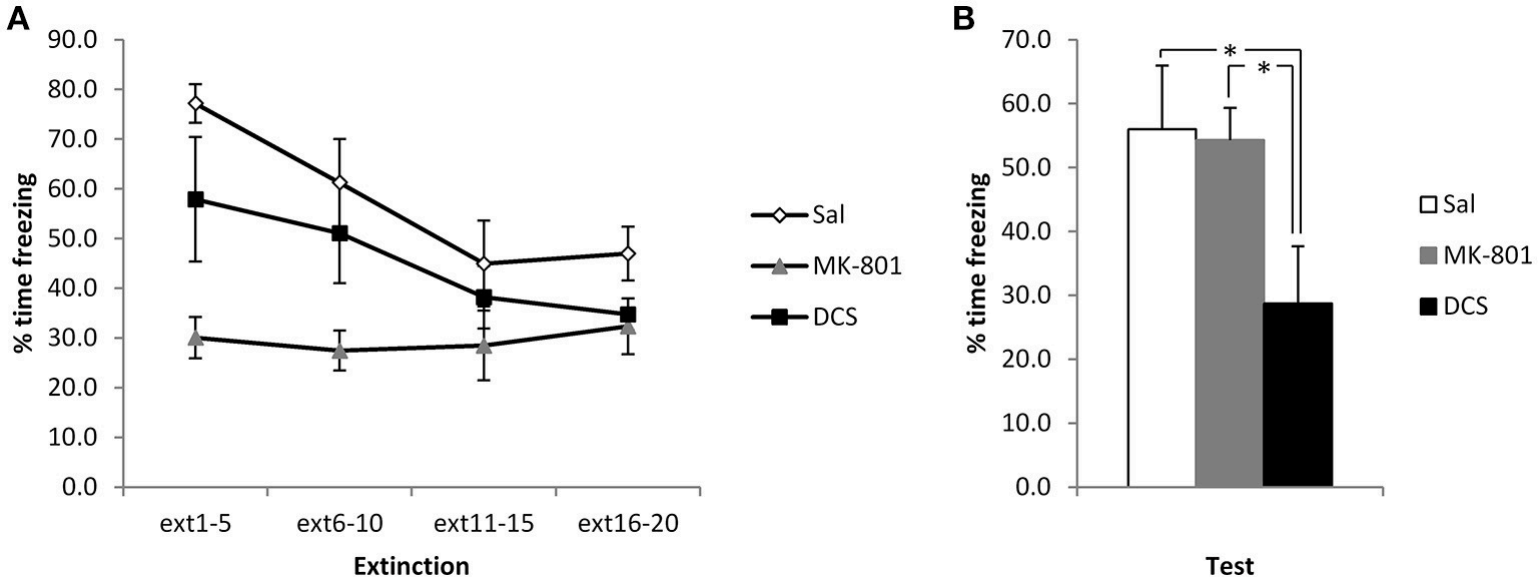
**Pre-extinction DCS reduces contextual freezing following strong conditioning**. MK-801 and DCS were injected 30 min prior to the extinction session. MK-801 acutely reduced contextual freezing during the first 2 bins of the extinction session **(A)**. DCS, but not MK-801, reduced contextual freezing at the subsequent test **(B)**. *N* = 8 per group. ^*^p < 0.05.

Again, to disambiguate extinction and reconsolidation accounts of the CBD-induced reduction in freezing, we injected MK-801 and DCS in the same stronger conditioning setting. Injection of DCS appeared to potentiate the extinction of contextual freezing, whereas MK-801 had no observable effect (Figure 4). ANOVA revealed a significant difference between the groups [*F*_(2, 21)_ = 5.31, *p* = 0.014, η^2^_*p*_ = 0.34, BF_10_ = 4.5], with *post-hoc* comparisons (*p* < 0.05) showing that the DCS group displayed lower levels of contextual freezing than both the Saline control group and the MK-801-injected rats (the latter two groups not differing from each other). This difference at test was not attributable to pre-existing differences at the extinction session, as the effect of group [*F*_(2, 21)_ = 6.27, *p* = 0.007, η^2^_*p*_ = 0.37, BF_10_ = 7.8) and group x bin interaction [*F*_(4.0, 42.4)_ = 3.78, *p* = 0.01, η^2^_*p*_ = 0.27, BF_10_ = 12.1] were both driven by lower freezing in the MK-801-injected rats. Analyses of the simple main effects of group at each bin revealed significant effects in the 1st and 2nd bins only [*F*'s_(2, 21)_ > 5.25, *p*'s < 0.015, η^2^_*p*_'s > 0.33, BF_10_'s = 4.3], for which *post-hoc* pairwise comparisons confirmed that the MK-801 group was lower than the saline group, with no other differences. As with the CBD experiment, for these stronger conditioning parameters there was a significant reduction in contextual freezing over the course of the extinction session [*F*_(2.0, 42.4)_ = 11.8, *p* < 0.001, η^2^_*p*_ = 0.36, BF_10_ = 12.1], apart from in the MK-801 group.

## Discussion

Here we demonstrate that i.p. CBD and MK-801 injections 30 min prior to extinction training had a common effect of increasing subsequent contextual freezing when contextual fear conditioning was relatively weak. In contrast, CBD replicated the effect of DCS to reduce contextual freezing when conditioning was stronger. These effects of CBD were critically dependent upon the extinction training, as CBD alone had no effect upon subsequent contextual freezing. These results suggest that the long-term impact of CBD on extinction depends upon the prior conditioning experience.

Under the strong conditioning procedure, CBD reduced subsequent freezing by a similar extent to DCS (CBD: 29.5 ± 2.5; DCS: 28.7 ± 9.0). In contrast, MK-801 had no observable effect. Given that DCS has previously been shown to potentiate contextual fear memory extinction (Yamada et al., 2011), this suggests that CBD also enhances extinction. In contrast, and somewhat surprisingly, there was no evidence that MK-801 impaired extinction under the same conditions. While there was no non-extinction control in the MK-801/DCS experiment, the no-extinction control in the CBD experiment suggests that the 20-min extinction session did induce a meaningful reduction in contextual freezing. This is consistent with the literature indicating that DCS only potentiates extinction when there is significant reduction in fear induced by the extinction training itself (Bolkan and Lattal, 2014). Therefore, it remains unclear why MK-801 did not impair this extinction-induced reduction in freezing, especially as it did under the weaker conditioning procedure. Indeed, there was no visual indication of an extinction impairment, as might have been expected with a ceiling effect were the freezing reductions in control animals too modest to observe a significant impairment. Nevertheless, the common effect of CBD and DCS supports the interpretation that each treatment reduced freezing through a potentiation of memory extinction.

While the combination of drug treatment and extinction training to reduce freezing is certainly consistent with an enhancement of extinction, studies of memory extinction rarely rule out alternative interpretations based upon long-lasting direct effects of drug treatment on the test itself. For i.p. injections of DCS, its effect on contextual freezing was not shown to be extinction-dependent (Yamada et al., 2011). However, when infused into the hippocampus, DCS did not have long-lasting direct effects on contextual freezing in the absence of extinction training (Bolkan and Lattal, 2014). Moreover, i.p. DCS reduced contextual freezing only when given before a long extinction session, but increased freezing when the extinction session was shorter (Yamada et al., 2009), demonstrating that these bidirectional effects must be attributable to the nature of the extinction session. In contrast, prior demonstrations of CBD reducing contextual freezing have not shown the effects to be dependent upon the extinction training (Bitencourt et al., 2008; Do Monte et al., 2013). CBD has been shown to have long-lasting antidepressant effects, reducing hyperactivity in an olfactory bulbectomy model of depression in mice (Linge et al., 2016). While these effects were observed with a higher single dose of CBD (50 mg/kg c.f. 10 mg/kg used here), a long-lasting modulatory effect on locomotor activity would likely impact upon the conditioned freezing measure of fear used here. However, in the absence of extinction training, CBD had no impact upon contextual freezing 24 h later. Therefore, CBD likely does potentiate contextual fear memory extinction.

The long-lasting reduction in contextual fear was preceded by an acute effect of CBD to reduce the expression of contextual freezing during the extinction session itself. This acute effect further supports the need for the aforementioned no-extinction controls, in order to rule out any direct persistent effects of CBD to reduce freezing. While CBD has been shown to reduce contextual fear memory expression (Resstel et al., 2006; Lemos et al., 2010) but see (Marinho et al., 2015), in the previous studies of contextual fear memory extinction CBD did not have an acute effect when it did potentiate extinction (Bitencourt et al., 2008; Do Monte et al., 2013). These discrepant effects might be related to the route of administration. Prior extinction studies have infused CBD directly into the infralimbic cortex (Do Monte et al., 2013) or intracerebroventricularly (Bitencourt et al., 2008), whereas the acute fear-reducing effect of CBD appears to involve the prelimbic cortex (Lemos et al., 2010), bed nucleus of the stria terminalis (Gomes et al., 2012), and perhaps the amygdala (Fusar-Poli et al., 2009). Therefore, while direct intracranial infusions dissociate the acute and long-lasting impacts of CBD, systemic injections appear able to induce both effects, presumably via actions in those dissociable neural loci. This is of translational relevance as both acute fear reduction and long-lasting enhancements in fear extinction have clinical benefits.

While the effects of CBD in our strong conditioning procedure are consistent with prior literature, the results from our weak conditioning experiments are less so. Under these conditions, the extinction session appeared to reduce contextual freezing to near baseline levels, such that while MK-801 impaired extinction to increase freezing, DCS did not further reduce freezing. Therefore, we might have expected CBD to replicate the effect of DCS (as it did in the stronger conditioning experiments), resulting in no modulation of contextual freezing. Instead, CBD replicated the effect of MK-801 to increase freezing at test. No-extinction controls confirmed that this elevation in freezing at test was not a direct effect of CBD, as might be predicted from a putative CBD-induced long-lasting attenuation of activity (Linge et al., 2016). Therefore, the effect of CBD is consistent with an impairment in memory extinction. The alternative explanation of an enhancement of reconsolidation is not consistent with the effects of MK-801 and DCS, nor is it supported by previous demonstrations that CBD impairs, rather than enhances reconsolidation (Stern et al., 2012). Why CBD should impair extinction when conditioning is weak is unclear. Although bidirectional memory modulatory effects with the same treatment have been observed previously (Suzuki et al., 2004; Lee et al., 2006), including with strength of conditioning being the only variable (Eisenberg et al., 2003), these have been interpreted as qualitatively similar effects on dissociable mnemonic processes (e.g., impairment of extinction to increase memory expression vs. impairment of reconsolidation to reduce memory expression). A bidirectional effect on extinction based upon the strength of the memory, to our knowledge, has not been previously reported.

There are two possible explanations for why the strength of conditioning should reverse the impact of CBD on extinction: Neuroanatomical competition and the differential stress levels evoked during extinction. In a series of studies of the impact of intra-medial prefrontal cortical infusions of CBD, Guimaraes and colleagues have previously demonstrated opposing effects of CBD depending upon the locus of infusion and levels of stress (Fogaça et al., 2014; Marinho et al., 2015). Intra-prelimbic infusions of CBD reduced the expression of contextual fear conditioning, whereas infusions into the infralimbic cortex increased contextual freezing. Given that our systemic injections of CBD here acutely inhibited contextual freezing during the extinction session following strong conditioning, these conditioning parameters (identical to those used in the infusion studies) might have biased the primary central locus of activity of CBD to the prelimbic cortex. However, while there has been no study to date of the impact of intra-prelimbic CBD infusions upon fear memory extinction, the neuroanatomical underpinnings of fear memory extinction implicates the infralimbic cortex, rather than the prelimic cortex (Sotres-Bayon and Quirk, 2010), consistent with the observation that intra-infralimbic CBD enhances contextual fear memory extinction (Do Monte et al., 2013). Therefore, while the neuroanatomical locus of effect (and indeed also the neurochemical and neurophysiological basis) of systemically-injected CBD in the current study remains to be determined, it is perhaps unlikely that our bidirectional results can be attributed to differential neuroanatomical loci of effects.

The same aforementioned studies of medial prefrontal cortical infusions also revealed apparent effects of stress levels upon the impact of CBD (Fogaça et al., 2014; Marinho et al., 2015). While prelimbic infusions of CBD reduced the expression of contextual freezing, they also increased measures of anxiety in the elevated plus maze (Fogaça et al., 2014). This anxiogenic effect is likely due to the relatively low stress levels induced in the elevated plus maze, as compared to contextual fear conditioning, as when testing was preceded by restraint stress to increase the stress levels present in the plus maze, the impact of CBD was reversed to reduce anxiety (Fogaça et al., 2014). For infralimbic infusions, the pattern of results was largely the reverse of those observed with prelimbic infusions, but restraint stress blocked, rather than reversed, the anxiolytic effect of CBD (Marinho et al., 2015). Although these results are related specifically to the acute expression of fear and anxiety, they reveal the possibility that the longer-term impact of CBD in relation to its effect on extinction might similarly be modulated by the stress levels present during the extinction session. One clear prediction would be that restraint stress prior to extinction following weaker conditioning would block the effect of CBD to impair extinction.

In summary, CBD had bidirectional effects on the extinction of contextual fear conditioning, depending on the nature of the initial fear conditioning. Nevertheless, in the more translationally-relevant stronger conditioning setting, CBD both acutely inhibited fear expression and enhanced extinction to produce longer lasting reductions in fear. These observations provide further support for the potential translational use of CBD in conditions such as posttraumatic stress disorder and specific phobias.

## Author contributions

CS and JL conducted the experiments and analyzed the data. CS, JL, CWS, and FG drafted the paper. All authors contributed to interpreting the original data, revising the draft and approving the final version of the paper.

## Funding

This research was funded by a FAPESP-University of Birmingham-University of Nottingham pump-priming award (JL, FG, CWS). The funders had no other involvement in any aspect of this work.

### Conflict of interest statement

FG is co-inventor of the patent “Fluorinated CBD compounds, compositions and uses thereof. Pub. No.: WO/2014/108899. International Application No.: PCT/IL2014/050023”; Def. US no. Reg. 62193296; 29/07/2015; INPI in 19/08/2015 (BR1120150164927). The other authors declare that the research was conducted in the absence of any commercial or financial relationships that could be construed as a potential conflict of interest.
